# Risk Assessment of Workplace Violence Against Nurses: How Data Collection Methods Influence Results—A Swedish and Italian Cross-Sectional Study

**DOI:** 10.3390/nursrep16010007

**Published:** 2025-12-24

**Authors:** Nicola Magnavita, Maivor Olsson-Tall, Sergio Franzoni, Lucia Isolani

**Affiliations:** 1 Department of Safety and Bioethics, Università Cattolica del Sacro Cuore, Largo Francesco Vito 1, 00168 Roma, Italy; 2 Department of Health Sciences, University West, 46186 Trollhättan, Sweden; maivor.olsson-tall@hv.se; 3 Poliambulanza Hospital, Università Cattolica del Sacro Cuore, Via Trieste, 17, 25121 Brescia, Italy; sergio.franzoni01@icatt.it; 4 Occupational Health and Safety Unit, Public Health Department, Local Health Authority, Azienda Sanitaria Territoriale AST, 62100 Macerata, Italy; lucia.isolani@sanita.marche.it

**Keywords:** occupational stress, work ability, selection bias, recall bias, social desirability, secondary research, spot survey, health surveillance, health promotion, workplace

## Abstract

**Background/Objectives**: Workplace violence (WV) against healthcare workers (HCWs) is a major hazard all over the world. Prevention requires a reliable risk assessment. The rate of HCWs reporting a violent event varies considerably across multi-year retrospective studies compared to periodic surveys. We conducted a rapid observational study to demonstrate that data collection methods are more important than socio-cultural and healthcare organizational differences in determining the frequency of reported violence. **Methods**: In June 2025, in a cross-sectional observational comparison, we examined a total of 236 nurses divided into three groups: the first two were recruited online from Brescia (Italy) and Trollhättan (Sweden), while the third group was composed of Latium (Italy) nurses participating in a sleep health promotion program who answered the same questions on WV online. All the workers reported the frequency of violent incidents experienced in the previous 12 months using the Violent Incident Form (VIF), occupational stress using the Effort/Reward Imbalance questionnaire (ERI), and work ability via the Work Ability Score (WAS). **Results**: In the three samples, WV was correlated positively with stress and inversely with work ability (*p* < 0.01), while no significant difference was found between Italian and Swedish nurses in relation to the spot surveys. The nurses questioned directly about WV were significantly younger and reported significantly higher rates of physical aggression (28% vs. 5%, *p* < 0.001) and all forms of violence (73% vs. 20%, *p* < 0.001) than those questioned indirectly during the census of all the HCWs. In a multivariate linear regression model, the WV experienced and poor work ability were highly significant predictors of work-related stress (*p* < 0.001). Nurses who had experienced WV in the previous year had an increased odds ratio (OR = 8.94; Confidence Interval 95% = 4.43; 18.01) of reporting a state of distress. **Conclusions**: Experience has shown that specific questioning about violence—the commonest method used—encourages respondents to report violent events and may induce overreporting. This method also tends to involve younger workers who are more exposed to WV. On the other hand, prospective studies based on official reports may be influenced by underreporting. Monitoring WV during health promotion interventions included in occupational health surveillance could minimize both phenomena. Systematic studies and meta-analyses which rely mainly on “ad hoc” studies may be biased.

## 1. Introduction

Although workplace violence (WV) has only recently entered the age-old history of occupational medicine (the first scientific articles reporting damage to workers date back only to the late 1980s [1,2,3], and in most countries employers are still not required to prevent this occupational risk [4], it has rapidly achieved a role of vital importance in healthcare. This has occurred not only because healthcare workers (HCWs) are the most frequently affected by assaults [5], but also because the risk is pervasive, recurrent and difficult to predict [6]. WV can have serious effects on workers’ physical [7] and mental health [8,9,10]. Furthermore, it reduces job satisfaction [11,12] and work engagement [13,14], stimulates turnover intention [15,16,17] and impacts on productivity [18,19], thus compromising interpersonal relationships with patients and the quality of care [20,21,22].

The main aspects of the phenomenon are known to us from a copious series of cross-sectional and retrospective studies. WV is associated with distress [23,24], fatigue [25], sleep problems [26,27,28,29], eating disorders [30] and burnout [31,32,33,34,35]. Serious physical assaults may lead to the death of HCWs [36,37,38]. Repeated and persistent non-physical violence can also be seriously harmful and lead to workers abandoning the profession [39] or, in extreme cases, suicidal ideation and suicide [40,41,42]. Younger and more inexperienced HCWs suffer the most severe effects [43,44,45]. No professional sector is immune to violence [46,47,48,49,50,51,52,53], although certain departments, such as psychiatric services and emergency rooms, are more exposed [54,55,56,57]. WV does not have a clear gender prevalence [58], although males tend to be more exposed than women to physical violence in Eastern countries [59,60,61,62,63] and in Italy [64]. On the other hand, women often report higher rates of harassment than males [63].

Longitudinal studies have shown that the relationship between violence and stress is reciprocal. Workers exposed to violence experience distress and reduced social support, favoring exposure to violence in subsequent years [65,66]. The relationship between WV and work ability is also circular. Indeed, prolonged exposure to violence damages relational skills and worsens caregiving, thereby reducing the work ability of HCWs [67]. It has also been observed that HCWs with poor work ability, namely older or disabled persons who experience significant difficulty in carrying out their professional duties, are more exposed to violence than their healthier colleagues [68].

This consolidated evidence might lead us to believe we have a perfect understanding of the phenomenon and are therefore able to prevent it. Unfortunately, this is not entirely true. Studies on WV are subject to severe methodological problems which, if not structured correctly, profoundly distort the results. The first problem is the definition of violence. Although authoritative international bodies have proposed valid definitions (Table S1), the fact remains that workers do not share a univocal definition of what type of act, behavior, or situation constitutes an episode of violence. This is especially true for verbal violence, where the same phrase can be interpreted as harassing or even threatening by one individual, but of no relevance by another [69]. This different perception is of the utmost importance in medicine because it is quite clear that only those who perceive a certain situation as violent can then feel distressed due to it. For this reason, WV investigations are asked to provide a precise operational definition of what the researcher means by violence and to accompany retrospective reporting of violent behavior with a measurement of personal perception of stress at the time of the response, so as to ascertain whether the reported event is important for the worker’s equilibrium.

A second significant issue is the choice of method used to measure exposure to violence. To assess the risk of violence, healthcare organizations mainly use multi-year retrospective analyses of official reports submitted by workers. This rather slow method is complemented in the literature by cross-sectional surveys, which, in much shorter timeframes, reach large numbers of HCWs. These two methods provide profoundly different results for the prevalence of workers reporting WV, thus preventing us from accurately assessing the phenomenon. This unsatisfactory situation is evident when comparing studies conducted in the same country.

In Italy, the retrospective studies conducted in large healthcare organizations (all with emergency and psychiatric services) indicate that over a period of 12 months approximately two out of a thousand workers risk being victims of physical assault, and approximately one or two out of a hundred may be subjected to some form of WV (Table 1).

In the same country, cross-sectional studies obtained prevalence rates that were more than an order of magnitude higher than retrospective ones. In surveys reserved for workers employed in a single company, between 6% and 12% reported having experienced physical violence in the previous year, while in online studies with self-selection of respondents from the general population of HCWs, the prevalence of physical violence rose to over 30%. The annual prevalence of all forms of WV is reported by one-third to 88% of respondents, with rates approaching 100% in psychiatric and emergency services (Table 2).

Since all the studies were conducted in the same period and refer to national health service facilities in the same country, the different rates are clearly not an authentic expression of different levels of risk. We believe that underreporting significantly affects the results of retrospective studies, while spot studies suffer from overreporting. Furthermore, in our opinion only by interviewing all workers during workplace health surveillance examinations is it possible to identify which violent incidents have affected workers’ well-being. On the contrary, we believe that the general characteristics of WV cannot be modified by the survey method and that the latter only impacts the measurement of prevalence.

Having observed methodological gaps in assessing the prevalence of workers exposed to WV, we designed a study to compare different methods in online surveys. Workers may be explicitly asked to report their experiences of violence, or this topic may be included in a broader health promotion context. They may be completely unknown to the interviewer, or the interviewer may be the doctor supervising them and to whom they could/should report the consequences of the violence they have experienced. Furthermore, since cultural context may influence the perception of an event as violent, it is necessary to compare different national contexts.

In this study, without changing the characteristics of the phenomenon, we aimed to demonstrate how sample selection and the form of presentation can influence the prevalence of WV. To this end, we selected several questions that contain an operational definition of violence experienced in the previous 12 months and administered them to two self-selected online samples in Italy and Sweden. The operational definition of WV was necessary in order to control variability in the individual concept of violence. A Northern European country whose history, traditions, and healthcare organization differed considerably from Italy was chosen to highlight the sociocultural differences that could influence the study outcome. We compared the responses of the samples with those of a numerically equivalent group of nurses selected through a census of participants in a sleep health promotion project during their annual health surveillance in Latium (Italy).

The hypotheses underlying our investigation were the following:

1. WV is positively associated with occupational stress;
2. WV is inversely associated with work ability;
3. Stress is inversely associated with work ability;
4. Self-selected samples explicitly questioned about WV report higher rates of violence than those declared by all workers undergoing health surveillance.

Confirmation of the first three hypotheses, which were consistent with the literature, would demonstrate the validity of the epidemiological design. The fourth hypothesis was completely new and sought to investigate the reasons for bias related to the survey method. In brief, our thesis was that the three samples would retain the same characteristics (association of WV with stress and disability) but would yield a different prevalence.

An assessment of the true number of workers experiencing WV is essential in order to provide appropriate counseling measures for victims, plan and develop adequate action to combat violence, and monitor the effectiveness of such intervention over time.

## 2. Materials and Methods

### 2.1. Population and Design of the Study

In Italy and in European countries, workers who are exposed to occupational risks undergo health surveillance. Besides regular medical examination and tests aimed at preventing occupational diseases, this includes the development of health promotion programs. These programs, which our university organizes every year with different health objectives, include screening through questionnaires on the basis of which workers identified as possible cases are invited to undergo further tests and treatment on the national health service. Although participation in health promotion programs is voluntary, worker participation is always very high, exceeding 85% [97]. The 2025 program focused on sleep problems. Some of the questions addressed WV, occupational stress, and work ability.

The methodology used in promotional campaigns for occupational risk prevention was studied by third-year students of the Nursing Sciences degree program at the Catholic University of Brescia, where the first author teaches. One of the students, who is among the authors of this article (S.F.), presented his nursing degree thesis on the relationship between work-related violence, stress and work ability among hospital nurses in Brescia and those at the Trollhättan hospital where he had completed an internship. To carry out this project, he singled out the questions on violence, stress, and work ability from the module used for health promotion in Latium, Italy and administered these questions to hospital nurses from Brescia (Italy) and Trollhättan (Sweden). Nurses were invited by their respective health departments to participate and completed the questionnaire anonymously by clicking a link or using the QR code provided on the invitation. Data was collected using the SurveyMonkey© platform, the same one used by the Latium workers monitored by the first author. The survey, which was conducted in June 2025, was terminated when in accordance with a previously calculated sample size, enough responses had been received. In this way, two samples were obtained, each composed of 75 nurses. In the same month, 86 nurses in Latium who were participating in a health promotion intervention on sleep that included questions on WV, formed the 3rd group. The study was therefore observational and cross-sectional with two groups of 75 and one of 86.

### 2.2. Questionnaire

Experience of WV was measured using the first questions of the Violent Incident Form (VIF), an instrument created by Arnetz [98] to describe the type and consequences of violent episodes. Four items concerned physical violence, threats, harassment, and stalking. For example, “In the past 12 months, have you experienced a physical assault while at work?” Each question included a succinct explanation. Physical assault refers to an attack, which may involve may not involve the use of weapons and can potentially result in physical harm. A threat means the intention to inflict physical harm. Harassment refers to any bothersome or unwelcome behavior (including words, attitudes, and actions) that contributes to a hostile work environment. Stalking is defined as a pattern of behavior involving persistent requests, messages, phone calls, and other forms of unwanted contact that elicit feelings of annoyance, concern, or fear. The fifth question sought to determine the principal perpetrator of violence.

The short Italian [99] and English version [100] of the Effort/Reward Imbalance (ERI) questionnaire [101] was utilized to assess work-related stress. Effort was assessed through three questions, each with a score ranging from 1 to 4, yielding a total range of 3 to 12. In contrast, reward was evaluated using seven questions, with scores ranging from 7 to 28. Stress represented the proportional relationship between effort and reward (effort/reward imbalance, ERI). ERI values higher than 1.0 indicate a state of distress. The reliability of effort, measured by three items, was 0.770 (0.794 in group 1; 0.680 in group 2; 0.790 in group 3), while the reliability of reward was 0.752 (0.805 in group 1; 0.711 in group 2; 0.784 in group 3).

Work ability was measured using the Italian [102] and English version of the Work Ability Score (WAS), which is the first question of the Work Ability Inventory (WAI) [103]. This one-item measure is expressed as a value ranging from 0 to 10 and has convergent validity with the total WAI score [104].

Overall, the questionnaire administered to nurses on violence/stress/work ability, both in the Italian and English versions was composed of 19 questions and took approximately 2 min to answer. We decided to use the original English version of the questionnaires instead of the Swedish translation because of the excellent level of knowledge of this language among Swedish nurses. The sleep health questionnaire used in Latium included a larger number of questions and took an average of 14 min to answer.

### 2.3. Ethics

The study protocol of the health promotion campaign “Sleep Health 2025”, containing the questions on violence, stress and work ability, was conducted in accordance with the Declaration of Helsinki and approved by the Territorial Ethics Committee 4 of Latium, Italy on 29 January 2025 (ID 5/2025). The research was then approved by the Committee for Good Research Practice and Ethics, University West, Trollhättan, Sweden on 16 May 2025 (ID F 2025/150). The workers signed an informed consent form and authorized the anonymous use of the data in their personal health document, also for scientific purposes. The research was not funded. The collected data are stored in a public repository and are freely accessible.

### 2.4. Statistics

To estimate the minimum sample size of Swedish and Lombardy nurses to be contacted, we considered the rate of physical assault measured during health surveillance of the nurses in Latium. We based the sample size calculation on physical violence (“an attack that… has the potential to result in physical harm”) because this type of violence is more universally recognizable than other forms of violence (threats, harassment) which depend on the victim’s perception and sensitivity. In this cohort, which has been continuously monitored for over 20 years [105], 5.0% of workers who were examined in the first quarter of 2025 reported having experienced at least one physical assault in the previous 12 months. The formula we have adopted for determining sample size was the following:

N = z^2^ × p (1 − p)/ε^2^ (1)

where we set the margin of error (ε) at 5%, the population proportion (p) at 5%, the confidence level score (z) at 95%. Consequently, using an automatic calculation system (Calculator.net©) [106], we ascertained that a minimum of 73 observations were needed to obtain a confidence level of 95%. We therefore stopped collecting online questionnaires from Brescia and Trollhättan as soon as 75 valid responses had been received. However, since health surveillance in Latium is a survey of all workers, we selected the nurses who agreed to participate in the same month as the other samples, thus obtaining 86 observations. Given the brevity of the questionnaire, we eliminated cases who responded to fewer than 18 of the 19 questions. After collecting responses online, we discovered that three Italian nurses and one Swedish nurse had provided only personal data without answering the questionnaires. These individuals were excluded from the study.

The collected variables were analyzed using the Kolmogorov–Smirnov and Shapiro–Wilk tests to determine whether the distribution was parametric or nonparametric. The sample size indicated that parametric methods were applicable despite the ordinal nature of the variables. Consequently, we examined the relationship between the variables by computing both Pearson’s r and Spearman’s rho.

An evaluation of the prevalence of disorders was integrated with that of the degree of uncertainty in prevalence (95% Confidence Interval, 95%CI) calculated by means of the Clopper–Pearson exact binomial test. Categorical data were compared using Pearson’s chi-square and Fisher’s exact test. Means were compared using ANOVA and post hoc Bonferroni comparisons. The correlation between variables was tested using Pearson’s r and Spearman’s rho. The relationship between violence and work ability on the perceived stress score was assessed using simple linear regression. A multivariate logistic regression model was used to calculate the risk of reporting a state of distress (defined as ERI > 1), by entering gender, age, WAS and WV as predictors.

Prior to examining the interactions among the variables through multiple linear regression, we ensured that these variables were not excessively correlated, as such relationships could compromise the integrity of the regression models. We calculated the variance inflation factor (VIF) to assess the interrelationship among predictor variables in a regression model. As in all cross-sectional studies, the regression equations do not imply a judgment about the causality of events.

All tests were performed using the 30.0 version of the IBM/SPSS statistical package (IBM Corp.: Armonk, NY, USA).

## 3. Results

Of the two hundred and thirty-six (236) nurses who took part in this survey, most were female (190, 80.5%). No gender differences were observed between the three groups (Table 3). The ages of the nurses from Brescia and Trollhättan who spontaneously responded to our invitation to participate, were very similar. Both groups were much younger than the nurses from Latium who had been contacted online during the workplace health surveillance (Table 4). The average age difference was more than ten years (*p* < 0.001).

A comparison of the violence nurses reported experiencing in the previous 12 months failed to highlight any difference between Brescia and Trollhättan. In contrast, the workers in Latium, who answered questions about violence during the sleep health promotion program, reported significantly lower rates of violence (*p* < 0.001) for all types of WV except stalking (Table 5).

In the two groups questioned directly about WV, physical violence was reported by 42 of the 150 respondents, corresponding to a 0.280 rate with a 95% confidence interval (95%CI) between 0.210 and 0.359. In the Latium sample, physical violence affected 0.047 (95%CI 0.013; 0.115) of the 86 participants. Similarly, in the two self-selected Italian and Swedish groups, the rate of threats was 0.360 (95%CI 0.283; 0.442), and that of harassment 0.560 (CI 95% 0.477; 0.641). In the Latium sample, 0.047 (CI 95% 0.013; 0.115) had experienced threats and 0.174 (CI 95% 0.101; 0.271) had undergone harassment. The overall rate of violence, according to the self-selected samples, was 0.727 (95%CI 0.648; 0.796), while it was 0.198 (95%CI 0.120; 0.298) among nurses under surveillance.

The levels of work-related stress and work ability reported by the two self-selected samples were very similar, while those reported by the nurses interviewed in Latium were much lower. Nurses from Brescia and Trollhättan reported levels of effort, reward, and work ability that did not differ significantly. The average level of perceived occupational stress was slightly higher among nurses from Brescia than among those from Trollhättan. In both samples, most workers reported a level of stress much higher than the theoretical level of equivalence between effort expended at work and rewards obtained. In Brescia, 82.7% of nurses were distressed, whereas the percentage of nurses in a state of distress in Trollhättan was 74.7%. In contrast, in Latium, a sample of nurses drawn from the entire population and interviewed in the same month of June, mostly reported acceptable levels of stress; only 16.7% reported excessive stress levels (Table 6). Overall, therefore, there were no significant differences between the Italian and Swedish samples recruited through a direct online survey on violence, while the picture drawn from the workers routinely interviewed during health surveillance indicated lower levels of violence, stress and inability.

In the 236 observations, males reported significantly greater exposure to threats than females (males 41.3%, females 20.5%, *p* = 0.003), but no significant gender differences were observed for physical violence (males 19.6%, females 19.5%, *p* = 0.989), harassment (males 43.5%, females 41.6%, *p* = 0.815), or for all forms of violence (males 65.2%, females 50.5%, *p* = 0.073).

The mean age of those who reported physical assaults was significantly younger than that of the other participants (35.41 ± 10.45 vs. 42.59 ± 12.15, *p* < 0.001). Similarly, those who had experienced threats (36.5 ± 10.55 vs. 42.87 ± 12.26, *p* < 0.001), those who had experienced harassment (37.93 ± 11.37 vs. 43.55 ± 12.28, *p* < 0.001) and all forms of violence (37.31 ± 11.26 vs. 45.65 ± 11.76, *p* < 0.001) had a lower average age than others. No age difference was observed among those who reported stalking (42.50 ± 12.27, *p* = 0.681).

**Figure 1:**
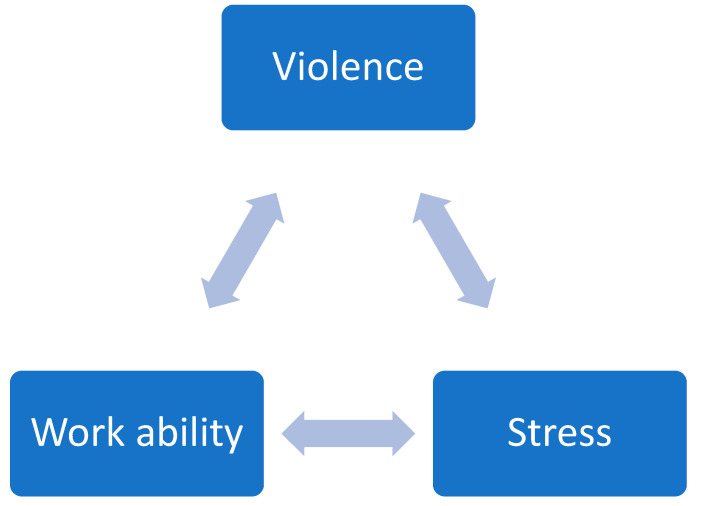
Relationship between workplace violence, work ability, and work stress.

Overall, the level of violence experienced was positively correlated with work-related stress and negatively correlated with work ability. Stress was also negatively correlated with work ability (Table 7). The bivariate correlation between these three variables is expressed in Figure 1.

After checking for multicollinearity among the variables of interest, we assessed the association between WV, stress, and work ability. In a multivariate regression model adjusted for age and gender, with stress (ERI) as the dependent variable, violence experienced and poor work ability were found to be significant predictors of perceived stress level. The model explains over a third of the individual variation in perception of work-related stress (adjusted R^2^ = 0.382) (Table 8).

We then evaluated the impact that WV has on the perception of stress. Using a logistic regression model adjusted for age and gender, with distress (ERI > 1.0) as the dependent variable, violence experienced and poor work ability were found to be significant predictors of the state of distress. Workers who had experienced some form of violence in the previous year had an almost nine-fold increased risk (OR = 8.94) of being distressed at work. Work ability was a protective factor. The model explained about half of the individual variance in distress (Nagelkerke R^2^ = 0.446) (Table 9).

## 4. Discussion

This study confirmed that WV is a significant problem for nurses in both Italy and Sweden. All our a priori hypotheses were confirmed. In all surveyed samples, WV was positively correlated with occupational stress and negatively correlated with work ability, as is extensively demonstrated in the literature [107,108,109]. Stress and work ability were inversely correlated. The three samples confirmed the bivariate relationships between WV, stress and work ability described in the literature. In the multivariate regression model, occupational stress showed higher values in nurses experiencing violence and insufficient work ability. In the logistic regression model, violence was associated with an 8.9 times higher risk of distress. The correspondence of our findings with the literature indirectly confirmed the validity of the responses given by nurses in the three surveys and demonstrated that the respondents had directly experienced the effects of WV. In agreement with the literature, the frequency of violent behavior failed to show a clear gender differentiation. Our study also confirmed the greater frequency of violent behavior against younger workers. This is observed in the literature and probably linked to being assigned tasks for which they are not yet fully trained [43,68]. The greater propensity of young people to participate in online surveys may be one reason why spot surveys obtain higher prevalence rates of WV than studies conducted on the whole population. The inability to verify the claims of online studies that are not followed up by medical examination may be another factor in inappropriate reporting.

Given the literature, our results were to be expected. The fourth hypothesis, namely that the degree of reported violence depends on the way the request is made, requires discussion. Our study has shown that the responses of the nurses from Brescia and Trollhättan were very similar, despite the considerable differences in the Italian and Swedish healthcare systems: the former is characterized by major inter-regional disparities, the latter by difficulties of access in rural areas, although the main difference is between the Italian socio-cultural structure that is closely linked to the family and the Swedish system that tends to separate work from private life. On the other hand, there was a significant difference in all parameters between the nurses in Brescia and Latium, all of whom belonged to the same healthcare system. Our hypothesis is that such a marked difference between data from two groups of Italian nurses can be explained only by the different way of presenting identical questions. The literature consistently indicates that WV episodes are severely underreported by workers, and this is certainly true for retrospective studies based on official reports. Cross-sectional studies, however, may be exposed to the risk of overreporting. We designed this study to demonstrate how these phenomena—under- and over-reporting—are closely linked to the methods used to administer questions and select the sample.

### 4.1. Causes of Under- or Over-Reporting

Studies on this topic generally conclude that violent incidents are severely underreported by HCWs [110]. There are numerous explanations for underreporting [111]. The most common is that HCWs view aggression by patients with cognitive impairment as part of their job [112,113,114,115] and are reluctant to report the patient’s behavior, partly because they are caught in an ethical conflict between providing help and correcting the patient’s behavior. HCWs who often rely on their professional ability to predict and prevent violence and believe that the occurrence of an assault is to some extent linked to their own behavior [116]. Qualitative studies have provided a list of other causal factors, broadly classified into organizational challenges, cultural and social barriers, and personal and emotional barriers [117], ranging from fear of retaliation [118] to the belief that reporting is futile because no one takes action to punish the perpetrators or change the conditions that foster violence [119,120,121,122]. Due to this complex set of factors, experience has shown that only major events involving a prognosis lasting several days are reported to the insurance company. A larger number of incidents are generally reported to colleagues, mainly so that they can implement precautionary measures, but no formal report is made to the health authorities. Consequently, many cases are unreported. This is a well-known phenomenon [69,123,124], and many measures have been proposed to reduce its extent [125,126,127].

On the contrary, the opposite phenomenon, overreporting, also occurs in the workplace [128]. Investigations specifically aimed at revealing the existence of WV and its consequences inevitably encourage workers to report events that occurred in the past. Legitimate concerns for their safety and the hope that better organizational procedures and measures can control the phenomenon induce the social desirability of a positive response from HCWs and can lead to bias whereby remote incidents can be mistakenly placed in a more recent time. Estimates of the frequency of violent events may therefore vary, depending on the method used to assess them. This could have an important impact on prevention measures, because understanding the risk is the first step in managing it.

Let us therefore consider the data available concerning the frequency of WV against HCWs, first of all in Italy, and then examine a summary of studies from other countries, paying attention to the methods of collection.

### 4.2. Retrospective Studies

Retrospective studies are conducted by collecting all complaints filed by workers seeking recognition of sick days following assaults, together with official reports forwarded to company services, such as Clinical Risk Management or the Prevention and Protection Service.

The retrospective analyses reported in Table 1 indicate that with prevalence rates ranging from 0.18% to 0.25% HCWs are exposed to physical violence, while with rates from 0.93% to 1.92% they are exposed to all forms of WV. Three recent studies were conducted in health companies situated close to the one we observed [72,73,77]. In each of these studies, assuming that each worker had been assaulted only once in the observation period, it was possible to calculate on the basis of the cases reported and the total number of employees in the company the percentage of workers assaulted annually. Given that differences in the way hospitals record incidents can influence the final result [129], we can see that these types of studies produced annual rates of physical violence of approximately 0.2 percent, and of just over 1% for all types of assault, which is equivalent to saying that physical assaults affected only one worker in 500, and that 99 out of 100 had no problems with harassment or incivility from patients, visitors, and colleagues—clearly, a situation too good to be true. Similar results have been obtained by other Italian hospitals that have adopted this method [70,74,76]. Retrospective surveys over several years lead to prevalence rates of WV against HCWs of around 1% per year also in other countries, such as Türkiye [121]. All these studies indicate that retrospective analyses of official reports fail to include all the incidents since they intercept only the most serious ones and those that the worker deems appropriate to report. Some healthcare organizations have implemented organizational and training measures to reduce underreporting from HCWs, thereby achieving a moderate increase in rates. For example, the University Hospitals of Turin (Italy) calculated a WV rate of 1.92% annually between 2015 and 2017 [71], and the University of Insubria (Varese, Italy) recorded a 2.08% annual rate of workers experiencing WV [75].

### 4.3. Cross-Sectional Studies

Cross-sectional studies reported in Table 2 are more numerous than multi-year retrospective studies and reveal widely varying results: exposure to physical violence ranges from 6.1% to 46.6%, while exposure to various forms of violence is between 29.6% and 96.0%.

In many studies, social media have been used to request participation. For example, in an online survey conducted between May 2018 and March 2020, La Torre et al. found 10% of HCWs reported experiencing physical violence in the previous 12 months, and 47.1% reported verbal aggression [64]. Other online studies have published percentages ranging from 29.6% to 57.1% [83,88,89]. HCWs who respond to social media are generally younger than the population of nurses working in the NHS. In May 2021, in a cohort of HCWs mainly composed of young nurses recruited through an online web-based survey, Ielapi et al. estimated a 32.0% rate of physical violence, a 64.0% rate of psychological aggression, and an 88.2% rate of verbal aggression [84]. In cases where studies involving a large number of participants were conducted in a single hospital, the rates tended to be lower than those in which the sample was selected through social media; however, also in single hospital studies, the percentage of HCWs reporting WV in the previous year ranged from 36.1% [85], 40.2% [79] 43% [80], 45% [82], 46.5% [87], 48.6% [81], to 49.4% [78]. High rates of WV are also reported by students, who are definitely less exposed than workers. For example, 35.1% of nursing students in Milan reported having been assaulted [86]. Even higher rates of aggression were obtained from sectoral studies in psychiatric or emergency departments: the percentages of emergency room workers who reported experiencing assaults in the previous year ranged from 71.1% to 96% [91,92,93,94,96], while the rates reported in psychiatric services were between 27.2% and 91.5% [90,95].

### 4.4. Systematic and Meta-Analytic Studies

The differences in prevalence rates between retrospective and follow-up studies can largely be explained by the way in which the information was collected. The ‘one-off’ investigations that explicitly declare the aim of recording violence against workers encourage the latter to report all incidents of assault, including minor ones, thus determining a social desirability bias [130]. The worker will be motivated to report an event that may have happened some time before the previous 12 months, thus causing a chronological recall bias [131]. Moreover, when samples are self-selected, workers who have undergone violence will be especially motivated to respond, while those who have not experienced violence will avoid responding and this may cause a self-selection bias [132]. In online studies, respondents may differ from non-responders in age and electronic literacy. Both factors are related to WV exposure, and this may induce a selection bias [133]. Furthermore, given that different research groups obtain responses from social media, it is also possible that the same person responds to multiple surveys, reporting the same experience several times over. The cumulative effect of these methodological problems means that there may be significant overreporting in ad hoc cross-sectional studies.

Spot cross-sectional studies are much more common than prospective longitudinal studies or multi-year retrospective surveys. Systematic reviews with meta-analyses rely mainly on one-off studies that consequently yield higher rates than those obtained from formal reports. The risk of selection bias is enhanced in umbrella reviews which repeatedly take into consideration the same original studies recorded by different systematic analyses, thus causing exponential overreporting. This is clear in the systematic studies currently available. Only a few systematic studies note that there is wide heterogeneity across the study methodology, definitions and rates and therefore avoid obtaining meta-analytic data [134]. Most of the others take account of the studies together regardless of method, thus leading to a potential systematic bias in meta-analysis.

A systematic analysis that included studies conducted before the COVID-19 pandemic and encompassed 253 studies involving more than 331,000 individuals, indicated that 61.9% (95%CI 56.1% to 67.6%) of workers had reported exposure to some form of WV in the previous year, with 24.4% (95%CI 22.4% to 26.4%) experiencing physical violence and 42.5% encountering non-physical violence. Verbal abuse, occurring in over two-thirds of cases, constituted the predominant type of non-physical violence. This was followed by threats in one-third of cases and sexual harassment in 12.4% of cases [135]. Another meta-analytic study led to a 19.33% (95CI 16.49% to 22.53%) estimated rate of physical violence [136]. Notably, substantial variations were observed across different countries, working hours, and workplaces. In China, the estimated prevalence of physical violence was 13.7% (95%CI 12.2% to 15.1%), while that of all forms of WV was 62.4% (95%CI 59.4% to 65.5%) [137]. A nationwide study involving over 100.000 Chinese nurses found WV rates of 30.3%, in addition to a strong association with mental health problems [10]. A meta-analysis of studies on home care nurses indicated that they had an annual rate of 0.135 for physical violence and 0.515 for verbal violence [49]. As regards doctors, 69% (95%CI 58% to 78%) had been subjected to WV [138]. In 2019, in Iranian emergency services, Sahebi et al. estimated a 36.39% (95%CI 27.29% to 45.50%) rate of physical assaults and a 73.13% (95%CI 68.64% to 77.62%) rate of verbal violence [139]. Research on WV increased during and after the pandemic. Based on over 44,000 observations, Matta et al. estimated that the prevalence rate of HCWs experiencing WV during the pandemic was 51% [58]. During the initial phase of the pandemic, there was a significant reduction in violent incidents [105]. This was followed, however, by an increase from mid-pandemic to late pandemic, and then a gradual return to pre-pandemic levels [140,141]. After the pandemic, the pooled rate of physical violence against HCWs was estimated to be 17% (95%CI: 6% to 28%) [142]; 17% (95%CI 14% to 21%) [143]; 23% (95%CI 14% to 34%) [144]. The proportion of HCWs who had received threats in the previous year was estimated to be 30% (95%CI 11% to 52%) [144]. The annual rate of all forms of violence has been estimated at 72% among Chinese nurses [145], at 35% (95%CI: 29% to 42%) among US nurses [146], and between 45.6% and 90% in studies published between 2013 and 2023 [147]. In the emergency department, verbal abuse was estimated at 77% [148], and in psychiatric nurses at 78% (95%CI 65% to 88%) [149]. According to an umbrella review of meta-analyses, the average rate of physical violence against HCWs is 20.8% for physical violence and 66.8% for verbal violence [150]. Another umbrella review reported an overall violence rate among HCWs as high as 78.9% [151].

### 4.5. Studies Conducted Through Health Surveillance

In our study, nurses from Brescia and Trollhättan reported experiencing very high rates of physical aggression (28%), threats (36%), harassment (56%), and all forms of violence (73%) in the previous year. These values can be placed at the upper end of the values estimated by meta-analyses and umbrella reviews. In contrast, in the Latium sample, 4.7% of the nurses were affected by physical violence, 4.7% reported threats and 17.4% experienced harassment, with an overall rate of 19.8% of nurses reporting some form of WV in the previous year. These latter values were below the levels estimated by meta-analyses. They were, however, in line with annual WV rates assessed in the same health company over the previous 20 years [105]. The percentages of nurses experiencing WV in the previous 12 months differed significantly from those for all forms of WV except stalking that has different causes [152]. These results can be explained by the method used. In the Latium census of all workers undergoing health surveillance, WV was included in the multipurpose questionnaire. Therefore, even those who had not experienced assault in the previous year participated in this survey because the focus of the questionnaire was on sleep problems, not workplace violence. Workers were free to join the online questionnaire, but experience shows that participation in health promotion projects is very high, so the survey took on the character of a census. Moreover, contact with the occupational physician after completing the questionnaire made it possible to clarify the characteristics of the violent incidents, assess their severity, and plan any necessary counseling, prevention, and control measures. The regular annual medical examinations also enabled nurses to be very precise about the timing of the assaults and prevent them from reporting remote events since those incidents and their consequences had already been noted in their personal health records. Consequently, data collection during health surveillance eliminates not only the overreporting bias of cross-sectional surveys but also most of the underreporting inherent in official records and appears to be the most accurate method [153].

Confirmation of this hypothesis is given by the only Italian study conducted on workers undergoing health surveillance in a university hospital in Sicily in 2023. The latter reported a 2.51% rate of physical assaults and a rate of all forms of WV of 26.8% [154]. During health surveillance, workers report incidents of violence they deem significant, regardless of whether they have been officially reported or not, but correctly overlook minor problems that have had no impact on their physical and mental well-being. Experience shows that underreporting can also occur during health surveillance, either because the worker believes that assault by a patient with cognitive impairment should not be reported, or because they are afraid to disclose assaults suffered by colleagues and superiors. However, the occupational physician’s attention to the issue of WV can educate workers to report and prevent under- and over-reporting.

### 4.6. Perspectives

In conclusion, we can state that the marked difference in rates of nurses experiencing violence in different healthcare settings was due to the different methods used to select the samples and administer the questions. Although the items were the same for all respondents, in the Brescia and Trollhättan samples, the topic of “violence” was explicit. By requesting an online response and terminating the survey after the first 75 responses, we wanted to increase the possibility of bias. The respondents were younger and therefore more exposed to violence than their older colleagues. Most of the self-selected nurses had recently experienced violent incidents they wished to report. Apart from differences in the prevalence of nurses reporting WV, the three samples showed the same relationship between violence, work ability, and stress. The nurses from Brescia and Trollhättan who reported higher rates of VW showed higher levels of stress and lower work ability than the nurses from Latium who underwent health surveillance in the same month of June.

To the best of our knowledge, this study is the only one that has assessed WV experience in different countries using the same questions but different sampling and survey presentation methods. Besides confirming the importance of WV for perceived stress and work ability and thereby corroborating the need for intervention to prevent WV and mitigate its effects [155,156,157], this study highlights serious bias in meta-analysis and umbrella review studies that do not differentiate between multi-year retrospective analyses and spot cross-sectional studies. This study empirically confirms theoretical expectations that emerged from the analysis of the marked difference between retrospective and spot studies and from related synthetic analyses.

We believe this study could have important implications for the health surveillance of HCWs and the prevention of WV. First, it demonstrates that systematic reviews and meta-analyses cannot be automatically assumed to be effective WV risk assessments, as unfortunately occurs with many methods adopted by healthcare organizations [158]. WV risk assessment could be much more accurate if the information were collected by occupational physicians during the health surveillance of workers. Our study demonstrated that responses on violence, stress, and workability can be collected in a few minutes and do not represent an excessive burden for either occupational health services or workers. If these questions are included in a program to improve worker well-being related, for example, to investigations on sleep, diet, or physical activity, it is possible to avoid the social desirability of negative responses, typical of spot WV surveys. Furthermore, the worker undergoing a medical examination will be able to discuss with the occupational physician the issue of any violence he/she has experienced, obtain the necessary counseling and contribute to the activation of prevention measures for the benefit of all workers. The data collected systematically during health surveillance can effectively monitor WV and verify whether the preventive interventions that have been implemented are effective in responding to environmental pressures [55,105,153].

### 4.7. Limitations

Our study has these strengths, but also many weaknesses. First, it is important to consider that the purpose of the study was not to describe in detail the violence experienced by nurses in different European countries, but to demonstrate, through a rapid cross-sectional comparison of three samples, the impact that the choice of method has on the results. The limited observation period and the barely sufficient sample dimensions reduced the stability of the results, although the limited dimensions of the sample were chosen specifically to enhance the effect of self-selection and highlight the difference between sampling methods. Across the three samples, WV maintained the same correlation with stress and workability, demonstrating that the observations were sufficiently stable. Despite the numerous cultural and healthcare system differences, the disparity between the self-selected Italian and Swedish samples supported our belief that the way questions are asked and respondents are invited is crucial to the results.

The cross-sectional nature of observations prevented us from interpreting the causality of the associations observed, which could only be explained on the basis of previous longitudinal studies. Of the three samples, only the one collected from Latium during health surveillance enabled the occupational physician to verify the occurrence of the reported WV episodes, whereas no objective verification could be made for the violence reported anonymously online.

In Italy and other countries where employers are required by law to assess the risk of WV, the difficulty encountered by managers in monitoring violence has often led them to use non-validated algorithms fueled by extemporaneous observations and supported by meta-analysis studies or umbrella reviews [158]. We believe that entrusting the monitoring of WV to the health surveillance service would be the most rational, economical and effective solution to this problem as it would lead to early identification of high-risk conditions and subsequent appropriate intervention.

## 5. Conclusions

A comparison of countries has shown that violence is widespread and exhibits similar characteristics across different work environments. The inexplicable difference in WV rates measured within the same country demonstrates that data collection methods directly influence results. This experimental study suggests that an assessment of violence experienced by workers can be efficiently conducted by surveying all workers during health surveillance. If workers are asked to report incidents experienced in the previous year during a broad online medical interview, we can be sure that they will only report the incidents they consider truly important for their well-being. This will enable us to comprehend the true impact that WV has on health and allow us to allocate preventive resources more effectively. Moreover, periodic health surveillance can enable us to monitor the incidence of WV over time and assess the causality of its effects.

We are confident that other workplace health and safety sectors can apply our study method that requires minimal time commitment for workers but provides reliable data for occupational medicine and prevention services. The problem of violence in healthcare settings is too serious to base prevention efforts on literature rather than on an effective assessment of the actual situation and a discussion of possible solutions with those involved.

## Figures and Tables

**Table 1:** Percentage of Italian workers who have experienced workplace violence (WV) in the previous 12 months according to multi-year retrospective studies.

Author	Place (Observation Period)	Physical WV Rate	All Forms of WV Rate
Sossai et al., 2017 [70]	Polyclinic San Martino, Genoa (2012–2015)	0.18% ^1^	
Viottini et al., 2020 [71]	University Hospitals of Turin (2015–2017)		1.92%
Sacco, 2022 [72]	Local health authority Roma3 (2010–2014)	0.24% ^1^	
Di Prinzio et al., 2022 [73]	Bambino Gesù Children’s Hospital, Rome (2019–2021)	0.25% ^1^	1.20% ^1^
Mele et al., 2022 [74]	Polyclinic Hospital of Bari (2017–2020)		0.93% ^1^
Veronesi et al., 2023 [75]	University of Insubria, Como and Varese (2021–2022)		2.08%
Terranova et al., 2025 [76]	University Hospital of Padua (2020–2022)		1.52% ^1^
Bianco et al., 2025 [77]	Umberto I Polyclinic, Rome (2019–2023)	0.21% ^1^	1.70% ^1^

^1^ data calculated by dividing the reported number of events by the population of HCWs.

**Table 2:** Percentage of Italian workers who have experienced workplace violence (WV) in the previous 12 months according to spot cross-sectional studies.

Author	Place (Observation Period)	Physical WV Rate	All Forms of WV Rate
Zampieron et al., 2010 [78]	595 nurses, 2 hospitals, Padua (July 2006)	9.1%	49.4%
Terzoni et al., 2015 [79]	903 HCWs, S. Paolo hospital, Milan	11.5%	40.2%
Luciani et al., 2016 [80]	198 nurses, S. Gerardo Hospital, Monza	6.1%	43%
Guglielmetti et al., 2016 [81]	296 HCWs, Melegnano hospital, Milan (Aug–Sep 2013)	46.6%	48.6%
Ferri et al., 2016 [82]	745 HCWs, Modena and Reggio Emilia hospitals		45%
Firenze et al., 2020 [83]	4545 HCWs, online (Jul–Oct 2018)		51.5%
Ielapi et al., 2021 [84]	203 HCWs, online (May 2021)	32.0%	88.2%
Converso et al., 2021 [85]	300 nurses, Turin hospitals		36.1%
La Torre et al., 2022 [64]	3659 HCWs, online (May 2018–Mar 2020)	10.0%	47.1%
Ferrara et al., 2022 [86]	Nursing students, University of Milan	8.1%	35.1%
Brunelli et al., 2023 [87]	200 HCWs in immunization center, Udine (March–April 2022)		46.5%
Bagnasco et al., 2024 [88]	6079 HCWs, online (Jan–Apr 2021)		32.4%
Stufano et al., 2025 [89]	3259 HCWs, Apulia hospitals (Nov–Dec 2023)	18.3% to 41.4%	29.6% to 57.1%
Palumbo et al., 2016 [90]	162 psychiatrists, Apulia (Jan–Mar 2014)	27.2%	77.8%
Ramacciati et al., 2019 [91]	1100 emergency nurses, online (Jul 2016–Mar 2017)	15.5%	91.5%
Cannavò et al., 2019 [92]	323 emergency HCWs, Umberto I Polyclinic, Rome (Jun 2016–Feb 2017)		87%
Gravante et al., 2020 [93]	83 emergency nurses, 2 hospitals, Campania (April–May 2019)	21.7%	71.1%
Ferri et al., 2020 [94]	27 Emergency nurses, Modena and Reggio Emilia hospitals		96%
Bizzarri et al., 2020 [95]	164 Psychiatric Service HCWs, Bolzano (June–July 2017)	21.3%	91.5%
Zaboli et al., 2024 [96]	49 emergency HCWs, Merano hospital		>90%

**Table 3:** Distribution of groups by gender.

Group	Frequency N (%)	Male N (%)	Female N (%)	*p* ^2^
1. Brescia, Italy	75 (31.8)	14 (18.7)	61 (81.3)	0.888
2. Trollhättan, Sweden	75 (31.8)	16 (21.3)	59 (78.7)	
3. Latium, Italy ^1^	86 (36.4)	16 (18.6)	70 (81.4)	

^1^ Collected during health surveillance. ^2^ Pearson’s chi square test.

**Table 4:** Comparison of groups by age.

Group	Age (Mean ± s.d.)	*p* ^2^
1. Brescia, Italy	37.5 ± 11.8	1 vs. 3 < 0.001
2. Trollhättan, Sweden	37.1 ± 11.5	2 vs. 3 < 0.001
3. Latium, Italy ^1^	47.9 ± 10.2	3 vs. 1 < 0.001 3 vs. 2 < 0.001

^1^ Collected during health surveillance. ^2^ ANOVA and Bonferroni post hoc comparisons.

**Table 5:** Workplace Violence (WV) reported.

Type of Violence	1. Brescia, Italy N (%)	2. Trollhättan, Sweden N (%)	*p* ^1^	3. Latium, Italy N (%)	*p* ^2^
Physical	22 (29.3)	20 (26.7)	0.716	4 (4.7)	<0.001
Threat	27 (36.0)	27 (36.0)	1	4 (4.7)	<0.001
Harassment	39 (52.0)	45 (60.0)	0.324	15 (17.4)	<0.001
Stalking	6 (8.0)	4 (5.3)	0.513	4 (4.7)	0.645
All forms of WV	54 (72.0)	55 (73.3)	0.855	17 (19.8)	<0.001

^1^ Brescia vs. Trollhättan, chi square test. ^2^ All groups, Pearson’s chi square test.

**Table 6:** Comparison of stress and work ability.

	1. Brescia, Italy Mean ± s.d.	2. Trollhättan, Sweden Mean ± s.d.	*p* ^1^	3. Latium, Italy Mean ± s.d.	*p* ^2^
Effort	9.63 ± 1.99	9.15 ± 1.96	0.139	7.92 ± 2.02	<0.001
Reward	16.85 ± 3.73	17.79 ± 2.88	0.088	18.61 ± 3.41	0.005
ERI ^3^	1.44 ± 0.53	1.25 ± 0.40	0.017	0.80 ± 0.37	<0.001
WAS ^4^	6.00 ± 2.00	6.27 ± 1.66	0.395	7.14 ± 1.89	<0.001

^1^ Brescia vs. Trollhättan, Student’s *t* test. ^2^ All groups, ANOVA. ^3^ Effort/Reward Imbalance. ^4^ Work Ability Score.

**Table 7:** Bivariate correlations between workplace violence (WV), stress ERI), and work ability (WAS). Spearman’s rho values (upper triangle) and Pearson’s r (lower triangle).

Variables	WV	ERI	WAS
WV, all forms	1	0.553 *	−0.294 *
Stress (ERI)	0.513 *	1	−0.459 *
Work ability (WAS)	−0.289 *	−0.447 *	1

* Correlation is significant at the 0.01 level (2-tailed).

**Table 8:** Relationship between workplace violence (WV), work ability (WAS), and work-related stress. Multivariate linear regression model.

Variables	Beta	t	*p*
WV, all forms	0.445	7.500	<0.001
Work ability (WAS)	−0.317	−5.675	<0.001
Gender	0.027	0.511	0.610
Age	−0.038	−0.675	0.500
Adjusted R^2^	0.382		

**Table 9:** Logistic regression. Relationship between workplace violence, work ability, and distress.

Variables	Odds Ratio	95%CI	*p*
WV, all forms	8.94	(4.43; 18.01)	<0.001
Work ability (WAS)	0.69	(0.57; 0.84)	<0.001
Female gender	1.29	(0.54; 3.08)	0.565
Age	0.97	(0.95; 1.01)	0.089
Nagelkerke R^2^	0.446		

## Data Availability

Data were deposited on Zenodo DOI 10.5281/zenodo.17100742. (Available from 11 September 2025).

## Supplementary Materials

The following supporting information can be downloaded at: https://www.mdpi.com/article/10.3390/nursrep16010007/s1, Table S1: Definitions of workplace violence.

## Public Involvement Statement

No public involvement in any aspect of this research.

## Guidelines and Standards Statement

This manuscript was drafted against the STROBE checklist for cross-sectional observational studies, available at: https://www.equator-network.org/reporting-guidelines/strobe/ accessed on 18 May 2025.

## Abbreviations

The following abbreviations are used in this manuscript:

CI95%	Confidence Interval 95%
ERI	Effort/Reward Imbalance
HCWs	Healthcare workers
VIF	Violent Incident Form
WAI	Work Ability Inventory
WAS	Work Ability Score
WV	Workplace violence
