# Supplementary material for: Risk Assessment of Workplace Violence Against Nurses: How Data Collection Methods Influence Results—A Swedish and Italian Cross-Sectional Study
Source: Nurs Rep. 2025 Dec 24;16(1):7. doi: 10.3390/nursrep16010007 (PMC12844877; doi:10.3390/nursrep16010007)
Supplement: Supplementary file 1 [file nursrep-16-00007-s001.zip › nursrep-3935897-supplementary.pdf]

Table S1. Definitions of Workplace Violence.

Definition	Description	Source
Workplace violence	Any action, incident, or behavior that deviates from reasonable conduct in which a person is assaulted, threatened, harmed, or endangered in their work or as a direct result.	International Labour Office (OIL-BIT), Geneve, SWI, 2003. Code of practice on workplace violence in services sectors and measures to combat this phenomenon. MEVSWS/2003/11 http://www.ilo.org/wcmsp5/groups/public/@ed_protect/@protrav/@safework/documents/normativeinstrument/wcms_107705.pdf
Workplace violence	Incidents involving work-related abuse, threats or assaults among health workers including physical, sexual, verbal and psychological abuse and workplace harassment.	World Health Organization (WHO) https://www.who.int/tools/occupational-hazards-in-health-sector/violence-harassment
Workplace violence	The act or threat of violence, ranging from verbal abuse to physical assaults directed toward persons at work or on duty.	National Institute for Occupational Safety and Health (NIOSH), USA https://www.cdc.gov/niosh/topics/violence/default.html
Workplace violence	Any act or threat of physical violence, harassment, intimidation, or other threatening behavior that occurs at the work site	Occupational Safety and Health Administration (O.S.H.A.), USA https://www.osha.gov/workplace-violence/
Workplace violence	An act or threat occurring at the workplace that can include any of the following: verbal, nonverbal, written, or physical aggression; threatening, intimidating, harassing, or humiliating words or actions; bullying; sabotage; sexual harassment; physical assaults; or other behaviors of concern involving staff, licensed practitioners, patients, or visitors	The Joint Commission https://www.jointcommission.org/en/standards/r3-report/r3-report-30
